# Supplementary material for: Safety and Efficacy of IL-12 Plasmid DNA Transfection into Pig Skin: Supportive Data for Human Clinical Trials on Gene Therapy and Vaccination
Source: Int J Mol Sci. 2024 Mar 9;25(6):3151. doi: 10.3390/ijms25063151 (PMC10970569; doi:10.3390/ijms25063151)
Supplement: Supplementary file 1 [file ijms-25-03151-s001.zip › ijms-2768617-supplementary.pdf]

Supplementary Table S1. The parameters of complete blood count (CBC) with differential analysis. The range for blood parameters of the pigs from plasmid groups was based on the control group, as all the pigs were also included in other studies, which could intervene with the blood parameters. Depending on the values, the brackets are marked green if in the control range, yellow if decreased or red if elevated.

	Time of sampling	WBC (x 10^9/L)	RBC (x 10^{12}/L)	HGB (g/L)	HCT (L/L)	MCV (fL)	MCH (pg)	PLT (x 10^9/L)	MPV (fL)	Neut (%)	Lymph (%)	Eos (%)	Baso (%)
Control group	before the procedure	20.13	6.13	102.33	0.32	52.37	16.70	609.33	7.90	46.40	47.23	1.20	0.30
	1 day	17.36	6.09	102.00	0.32	51.90	16.80	472.33	8.37	37.90	51.87	5.23	0.17
	2 days	15.68	6.45	107.67	0.34	52.07	16.63	488.67	8.67	46.20	43.83	3.93	0.30
	3-5 days	19.31	6.85	114.00	0.36	52.00	16.55	662.00	7.45	44.60	46.90	2.20	0.30
	7 days	18.93	6.78	112.67	0.35	51.83	16.63	503.67	7.97	47.00	44.70	2.43	0.30
	14 days	21.54	6.26	101.50	0.32	51.45	16.30	663.50	8.25	46.50	45.70	2.10	0.20
	21 days	25.61	7.59	123.00	0.39	51.80	16.20	690.00	7.10	40.70	47.60	4.30	0.40
	28 days	16.22	6.38	103.00	0.31	48.50	16.10	758.00	7.30	34.90	57.20	2.00	0.20
1 mg/ml plasmid group	before the procedure	14.18	5.67	98.33	0.31	54.30	17.37	478.33	7.90	27.80	65.17	0.87	0.23
	1 day	15.12	5.15	88.00	0.27	53.47	17.07	427.33	8.40	37.93	53.83	3.47	0.20
	2 days	17.36	5.73	99.33	0.31	53.87	17.33	390.67	9.30	43.53	46.50	2.43	0.33
	3-5 days	16.42	5.53	95.00	0.29	53.33	17.20	376.33	8.63	32.77	57.33	2.17	0.27
	7 days	18.30	5.50	95.33	0.30	54.67	17.33	571.67	8.07	40.47	52.03	1.47	0.23
	14 days	20.90	5.88	100.50	0.32	54.35	17.25	564.00	8.15	41.65	51.60	1.40	0.25
	21 days	22.00	6.65	110.00	0.34	51.90	16.60	630.00	7.70	32.60	57.20	2.10	0.40
	28 days	22.31	5.18	86.00	0.27	52.80	16.50	487.00	8.60	47.40	47.00	3.20	0.20
2 mg/ml plasmid group	before the procedure	15.04	5.48	87.33	0.28	51.13	15.97	578.33	7.53	41.07	52.97	1.47	0.27
	1 day	18.97	5.48	85.00	0.27	49.00	15.50	420.00	8.65	43.95	47.55	3.40	0.25
	2 days	19.32	5.96	94.67	0.30	50.47	15.93	459.33	9.03	46.57	43.23	3.60	0.40
	3-5 days	17.57	5.91	94.00	0.30	50.43	15.93	371.33	7.90	37.93	52.37	3.47	0.47
	7 days	18.54	5.77	93.00	0.29	50.37	16.10	605.67	8.27	47.37	44.03	2.67	0.27
	14 days	19.90	5.72	92.00	0.30	51.20	16.10	476.50	8.30	46.50	45.45	2.15	0.25
	21 days	22.62	6.26	102.00	0.33	52.50	16.10	615.00	7.60	54.90	37.40	2.00	0.20
	28 days	16.31	5.43	87.00	0.28	51.30	16.00	634.00	7.30	50.20	42.80	1.10	0.10

Supplementary Table S2. The parameters of biochemistry analysis of coagulation factors (PT, prothrombin time, APTT, activated partial thromboplastin clotting time), electrolytes, glucose and blood urea nitrogen (BUN). The range for biochemistry parameters of the pigs from plasmid groups was based on the control group, as all the pigs were also included in other studies, which could intervene with the biochemistry parameters. Depending on the values, the brackets are marked green if in the control range, yellow if decreased or red if elevated.

	Time of sampling	PT (s)	APTT (s)	Sodium (Na) mmol/l	Potassium (K) mmol/l	Chloride (Cl) mmol/l	Calcium (Ca) mmol/l	Glucose (mmol/l)	BUN (mmol/l)
Control group	before the procedure	11.03	15.87	138.90	3.87	97.50	2.57	5.21	2.19
	1 day			144.10	4.06	99.97	2.35	5.39	2.80
	2 days			146.93	4.69	102.27	2.48	6.56	2.86
	3-5 days			142.65	5.14	96.45	2.56	5.88	3.37
	7 days	10.37	13.80	142.23	4.95	96.50	2.60	6.59	3.43
	14 days	10.95	15.85	141.05	4.82	98.50	2.60	6.54	4.35
	21 days	10.20	14.80	142.90	5.70	48.90	2.81	7.59	4.70
	28 days	10.30	14.60	140.20	4.44	94.70	2.58	4.64	2.77
1 mg/ml plasmid group	before the procedure	11.93	15.07	139.60	3.92	94.93	2.55	6.47	2.92
	1 day			143.87	3.84	99.07	2.30	5.87	3.84
	2 days			142.83	4.55	98.73	2.43	5.61	3.45
	3-5 days			141.33	4.87	99.17	2.47	6.54	3.47
	7 days	10.53	14.20	141.20	5.08	79.63	2.57	5.79	2.62
	14 days	11.50	16.20	141.35	4.75	96.75	2.59	5.57	3.73
	21 days	10.80	14.60	147.60	4.56	97.60	2.78	6.41	4.70
	28 days	11.10	15.10	134.20	3.78	92.50	2.17	2.85	3.62
2 mg/ml plasmid group	before the procedure	11.50	14.93	140.77	3.91	100.07	2.45	6.00	3.95
	1 day			143.83	4.04	98.37	2.36	5.50	4.70
	2 days			143.30	4.28	81.83	2.37	6.43	3.04
	3-5 days			141.83	4.58	100.10	2.44	6.64	3.03
	7 days	10.67	14.57	141.13	4.48	94.93	2.56	6.13	3.96
	14 days	11.30	15.50	141.75	4.26	99.30	2.59	4.76	3.39
	21 days	10.10	14.50	141.80	4.38	48.00	2.82	7.05	4.33

	28 days	10.70	15.40	141.50	4.13	100.10	2.50	4.78	3.35

Supplementary Table S3. The biochemistry analysis parameters were creatinine, inorganic phosphate, alkaline phosphatase (AP), alanine aminotransferase (ALT), aspartate aminotransferase (AST), total protein and albumin. The range for the plasmid groups was set from the control groups. Depending on the values, the brackets are marked green if in the control range, yellow if decreased or red if elevated.

	Time of sampling	Creatinin (umol/l)	Anorganic phosphat (mmol/l)	AP (U/l)	ALT (U/l)	AST (U/l)	Total protein (g/l)	Albumin (g/l)
Control group	before the procedure	87.91	2.77	170.52	35.35	30.63	50.86	26.70
	1 day	82.21	2.91	167.82	54.22	90.34	50.33	26.33
	2 days	91.42	3.00	160.13	56.95	43.38	55.28	28.07
	3-5 days	71.09	3.36	126.32	54.67	55.18	63.67	30.47
	7 days	93.60	2.97	140.38	50.69	111.01	58.65	29.89
	14 days	96.21	3.07	109.47	38.23	33.96	60.60	29.48
	21 days	111.32	3.40	140.98	49.91	52.27	68.50	32.76
	28 days	100.28	3.61	136.42	39.09	30.12	57.35	28.15
1 mg/ml group	before the procedure	97.88	2.93	147.82	52.49	41.09	54.02	29.42
	1 day	86.74	2.94	142.59	63.29	122.78	47.74	26.22
	2 days	92.97	3.04	120.90	64.47	54.53	48.37	26.27
	3-5 days	94.13	3.00	106.22	59.35	36.20	50.22	26.93
	7 days	98.11	3.05	109.11	59.47	54.41	56.31	28.65
	14 days	116.66	3.26	114.29	68.84	35.35	60.60	30.49
	21 days	140.90	3.37	115.20	89.01	38.53	67.85	31.24
	28 days	119.84	2.89	98.56	66.02	45.84	57.38	25.48
2 mg/ml group	before the procedure	91.83	2.86	155.96	54.57	39.30	52.15	24.31
	1 day	115.85	3.10	144.93	63.94	77.72	46.95	23.45
	2 days	89.09	2.81	129.14	66.37	35.39	51.82	25.10
	3-5 days	91.58	2.85	120.09	63.50	27.73	54.60	25.71
	7 days	94.58	2.92	120.28	65.91	29.60	57.11	26.83
	14 days	106.80	3.04	95.91	57.35	31.50	61.38	28.09

	21 days	93.41	2.98	96.21	68.67	31.30	59.46	30.05
	28 days	89.26	3.54	111.00	67.60	28.59	60.62	29.94
